# A Canadian Weekend Elective Pediatric Surgery Program to Reduce the COVID-19–Related Backlog: Operating Room Ramp-Up After COVID-19 Lockdown Ends—Extra Lists (ORRACLE-Xtra) Implementation Study

**DOI:** 10.2196/35584

**Published:** 2022-03-15

**Authors:** Clyde Matava, Jeannette So, RJ Williams, Simon Kelley, ORRACLE-Xtra Group

**Affiliations:** 1 Department of Anesthesia and Pain Medicine Hospital for Sick Children Toronto, ON Canada; 2 Perioperative Services Hospital for Sick Children Toronto, ON Canada; 3 Division of Orthopedic Surgery Hospital for Sick Children Toronto, ON Canada; 4 Department of Surgery University of Toronto Toronto, ON Canada; 5 See Acknowledgments

**Keywords:** waiting lists, quality improvement, patient satisfaction, COVID-19, ambulatory surgery, pandemics, Canada

## Abstract

**Background:**

The COVID-19 pandemic caused by the SARS-COV-2 virus has resulted in unprecedented challenges for the health care system. A decrease of surgical services led to substantial backlogs for time-sensitive scheduled pediatric patients. We designed and implemented a novel pilot weekend surgical quality improvement project called Operating Room Ramp-Up After COVID Lockdown Ends—Extra Lists (ORRACLE-Xtra).

**Objective:**

Our overall goals are to increase patient access to surgery (and reduce the wait list), improve operating room efficiencies, and optimize parent and staff experience.

**Methods:**

Using the DMAIC (define, measure, analyze, improve, control) framework, we implemented ORRACLE-Xtra in a tertiary care academic pediatric hospital during a quiescent period of the COVID-19 pandemic. We defined process and outcome measures based on provincial targets of out-of-window cases. Parental and staff satisfaction was tracked by surveys.

**Results:**

ORRACLE-Xtra led to 247 patients receiving surgery during the pilot period, resulting in a 5% decrease in the total number of patients on our wait list with Paediatric Canadian Access Targets for Surgery IV (147/247, 59.5%), with 38.1% (94/247) out-of-window of provincial targets. Most of the process and outcome measures were met or exceeded. Overall parental satisfaction was at 95.8% (110/121), with 79% (64/81) of staff reporting satisfaction with working weekends.

**Conclusions:**

Through the ORRACLE-Xtra pilot program, we have shown that hospitals impacted by COVID-19 can reduce the surgical backlog using innovative models of service delivery in a Canadian context. Sustained funding is critical to achieving more meaningful reductions in wait times for scheduled surgeries over the longer term and needs to be balanced with staff well-being.

## Introduction

The COVID-19 pandemic caused by the SARS-COV-2 virus has resulted in unprecedented challenges for the health care system. In March 2020, the Canadian Province of Ontario’s Ministry of Health (MoH) enacted a provincewide directive to decrease surgical services to preserve hospital capacity [1]. However, this directive led to significant backlogs for time-sensitive scheduled (elective) surgeries in adult and pediatric centers across Ontario with an impact on patients and families, similar to reports from other jurisdictions [2-5]. In children, surgical delays are a source of significant morbidity because of three key issues: timing of surgery has a critical impact on the growth and development of the child; treatable conditions may deteriorate over time because of the effects of growth; and excessive surgical wait times may result in the need to perform more complex surgeries than were initially planned, leading to an increase in avoidable complications [5-9].

Due to the mandated reduction in surgical activity, the surgical wait list at our institution increased by 29% from 3799 (December 2019) to 4915 patients by the end of December 2020. This increase was despite a successful increase in surgical activity from July 2020. The Paediatric Canadian Access Targets for Surgery (P-CATS) use diagnosis-based categories to define time-based targets (windows) for completing scheduled pediatric surgeries [10]. During the COVID-19 pandemic, between March and December 2020, PCATS-defined out-of-window rates for cases on our surgical wait list increased from an already high rate of 44% to 58%. Using novel machine learning algorithms, we demonstrated that even on resuming our usual level of surgical activity the surgical wait list would not decrease without a substantial increase in resources. In resource-constrained environments, more medically urgent procedures may gain preferential access to the operating room (OR), which leads to a virtual two-tiered wait list system where lower acuity day-case procedures have further increased in wait times. By December 2020, our out-of-window rates for day-case surgical procedures exceeded 60% and increased at a faster rate than those of higher complexity surgeries.

Following the resumption of scheduled care, the MoH provided additional targeted funding for 3 months (January to March 2021) to address the surgical backlog. At our institution, despite additional funding, limited health human resources and OR real estate prevented an increase in scheduled surgical activity hours during our typical Monday to Friday workweek. In addition, we calculated that any attempt to increase the proportion of day-case surgical activity during the weekday schedule would serve to overwhelm the limited postanesthesia care unit (PACU), causing bottlenecks. These bottlenecks would reduce OR access to more medically urgent and complex cases. Thus, we recognized that an innovative program would be required to increase surgical activity above historical norms, specifically target low-acuity day-case procedures, and ultimately reduce the surgical backlog. In response to the MOH funding initiative and respecting our human health resources and physical capacity limitations, we designed and implemented a novel pilot weekend surgical program called Operating Room Ramp-Up After COVID Lockdown Ends—Extra Lists (ORRACLE-Xtra).

This pilot QI project aimed to assess the feasibility of efficiently reducing the number of patients on the surgical wait list by scheduling elective surgeries on the weekends with a high level of satisfaction among participating parents and providers.

## Methods

### Objectives and Aims

We aimed to efficiently reduce patients on the surgical wait list by scheduling elective surgeries on the weekends. By opening six dedicated ORs for scheduled surgery on each of the 12 weekends between January 9 and March 28, 2021, we targeted a 5% reduction in the surgical backlog (250 children) based on previous modelling using machine learning predicting anesthesia and surgical times along with nursing staff availability. In addition, the entire weekend scheduled surgery program was considered entirely separate from the regular emergency surgical service that ran in parallel within the same OR complex.

The secondary aim was to evaluate the process and outcome measures we defined as integral to the program’s success and long-term establishment. Given that our institution had not previously performed scheduled weekend surgeries, this program also provided us with an opportunity to pilot novel processes that could potentially increase OR efficiencies during the typical weekday schedule. We aimed to compare process measures, including the number of scheduled cases completed, rate of same-day cancellations, rate of preanesthesia fasting violations, the proportion of on-time starts (8 AM and 8:15 AM), operational block use, and room turnover times. Finally, we aimed to assess caregiver satisfaction and provider satisfaction of participating in the weekend program.

The project was designed using the DMAIC (define, measure, analyze, improve, control) methodology of Lean QI. The DMAIC methodology is a Six-Sigma data-driven improvement cycle designed to identify and address inefficiencies in a process, improve process outcomes, and make these improvements more predictable over time [11-13]. We chose this technique as it allowed us to improve our processes rapidly and iteratively over the 12-week pilot phase.

### Ethic Approval

We obtained institutional approval from the Hospital for Sick Children for this quality improvement project (QIP-2021-01-08).

### Governance and Principles of ORRACLE-Xtra

The ORRACLE-Xtra steering committee was established and consisted of surgical, anesthesia, and OR nursing leadership who identified a series of guiding principles for the weekend program (Textbox 1) and patient selection criteria (Multimedia Appendices 1 and 2).

**Guiding principles and rationale of the Operating Room Ramp-Up After COVID Lockdown Ends—Extra Lists (ORRACLE-Xtra) pilot program.**

**Maximize reduction in the number of patients on the wait list**

Demonstrate beneficence and utility for efficient use of limited Ministry of Health funding

**Target surgical divisions with the largest wait list, including high numbers of day cares**

Demonstrate beneficence and utility for efficient use of the limited Ministry of Health fund

**Target out-of-window cases**

Aim for equity in case selection by prioritizing cases with longest waits as per standardized Paediatric Canadian Access Targets for Surgery–recommended wait times

**Low acuity surgeries**

Achieve high throughput per surgical list to minimize complex logistics and variability in case length, and reduce the likelihood of needing inpatient stay

**Clearly defined case selection criteria**

Equity in case selection, minimize day-of-surgery cancellations, increase operational efficiency

**Minimize the need for inpatient admissions**

Avoid increasing bed census over weekend in pandemic-related already resource-constrained environment, minimizing number of extra nursing staff required to run the weekend program

**Separate ORRACLE staffing and logistics from the regular weekend emergency surgical team (no crossover of resources)**

Reduce risk of scheduled surgery cancellations due to sharing or competing for same perioperative human health resources

**Minimize number of required specialist technical services and staff (eg, pathology)**

Ensure cost-effective program, minimizing the number of volunteer staff required to be present to run the weekend program

**Promote an efficient team-based approach (same operating room nurse, anesthetist, recovery nurse, and support staff per each operating room)**

Optimize surgical list efficiency and promote team well-being, camaraderie, and morale

**Staff free to go home when the last case has finished**

Provide an incentive for increased operating room efficiency

The steering committee met weekly and, using the DMAIC process, reviewed the operative cases, operational metrics, logistical challenges, and opportunities for improvement from the previous weekend (Multimedia Appendix 3). Due to pandemic restrictions on in-person meetings, we used a combination of online meetings and email for discussion, data analysis, and decision-making (Multimedia Appendix 4).

**Figure 1:**
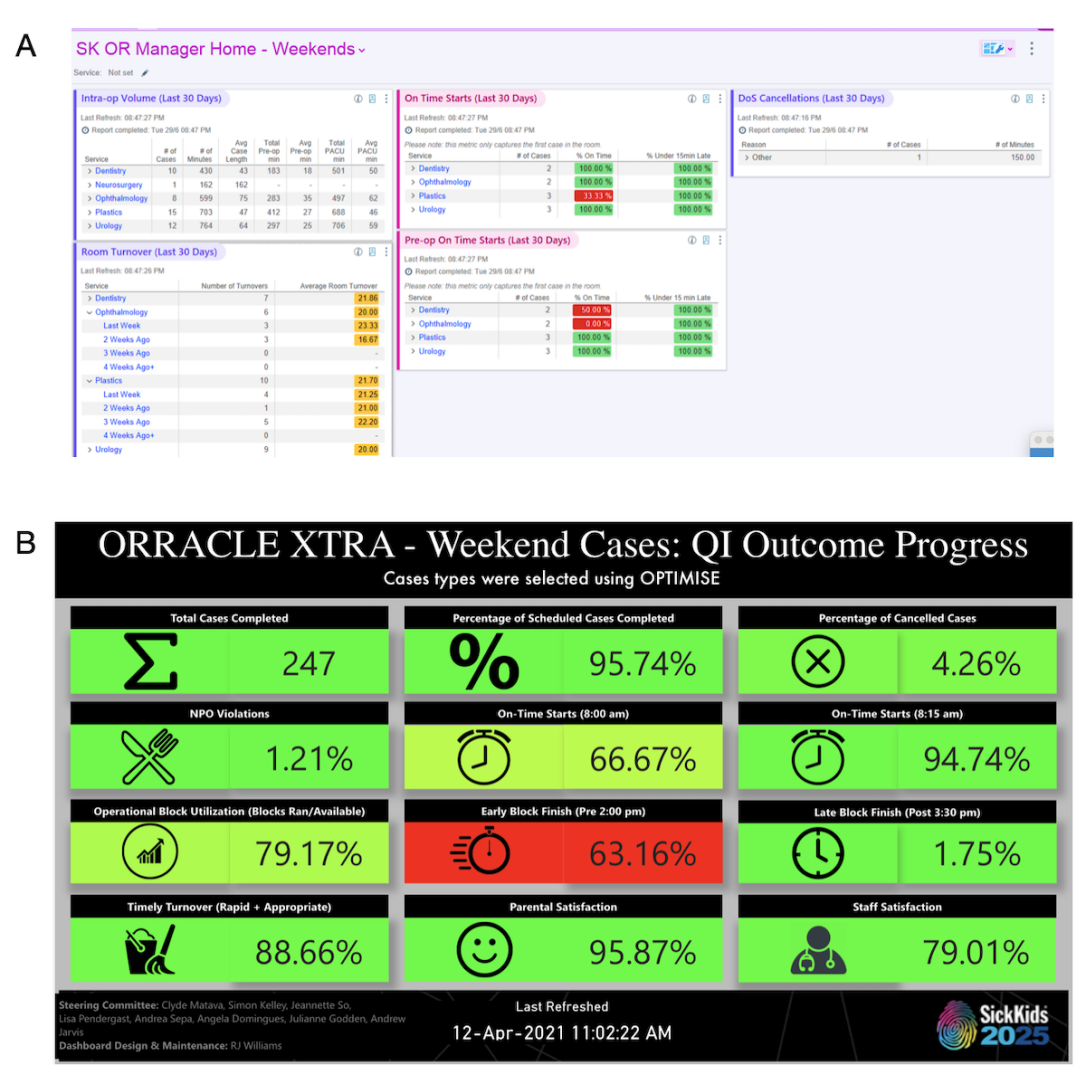
Dashboards used to inform data-driven decisions for Operating Room Ramp-Up After COVID Lockdown Ends—Extra Lists (ORRACLE-Xtra). Panel A shows the Epic dashboard depicting real-time patient and operational information. Panel B shows the PowerBI dashboard depicting ORRACLE-Xtra process outcome measures.

Following discussion and consensus, we identified process and outcome measures for the ORRACLE-Xtra program (Multimedia Appendix 5). To provide a data-driven approach as per DMAIC, we designed and implemented dashboards that summarized outcome measures in real time. We integrated the dashboards into our electronic health record system, Epic and Power BI (Microsoft Corporation). The Epic dashboard depicted patient health information linked to patient records, procedures, and date of surgery (Figure 1 and Multimedia Appendix 5). The PowerBI dashboards continually illustrated operational efficiency metrics such as OR start times, room turnover times, surgical case length, preanesthesia fasting violations, and tracked changes in the surgical wait list (Figure 1). We determined baseline using data from the prepandemic period. To collect caregiver and provider experience and satisfaction with the program, we used an online REDCap-based survey delivered by email within 24 to 48 hours after surgery [14].

### Staff Eligibility

Using regular department communication channels, we sent volunteer calls to anesthesiologists, nurses, surgeons, OR attendants, admission clerks, and equipment processing personnel to staff and support the weekend-scheduled surgical lists. As a result, we ran up to six ORs each weekend (three on Saturday, three on Sunday). Once we confirmed the required staff for each surgical list, we identified patients who met specific case selection criteria. All staff were compensated for their time.

### Patient Eligibility

Patients suitable for the weekend program were identified from the existing surgical waitlist using clear selection criteria developed by the steering committee (Multimedia Appendix 2). The overarching principle used was to identify medically stable patients who required low-complexity short surgical procedures. These patients could be discharged home from the PACU without requiring an inpatient bed. In addition, these patients should have long surgical wait times as defined by P-CATS and the provincial out-of-window status. The case selection criteria were distributed to each surgical service to assist with patient selection.

### Data Analysis

Using the Canadian Pediatric Perioperative Outcomes National Datalake dictionary, data was extracted from Epic and imported into Excel (Microsoft Corporation) for analysis. We used descriptive statistics to summarize the data. Continuous variables were depicted as mean (SD) values and categorical variables as numbers (proportions). This quality improvement project is reported according to the SQUIRE 2.0 (Revised Standards for Quality Improvement Reporting Excellence) checklist [15].

## Results

### Demographics

**Figure 2:**
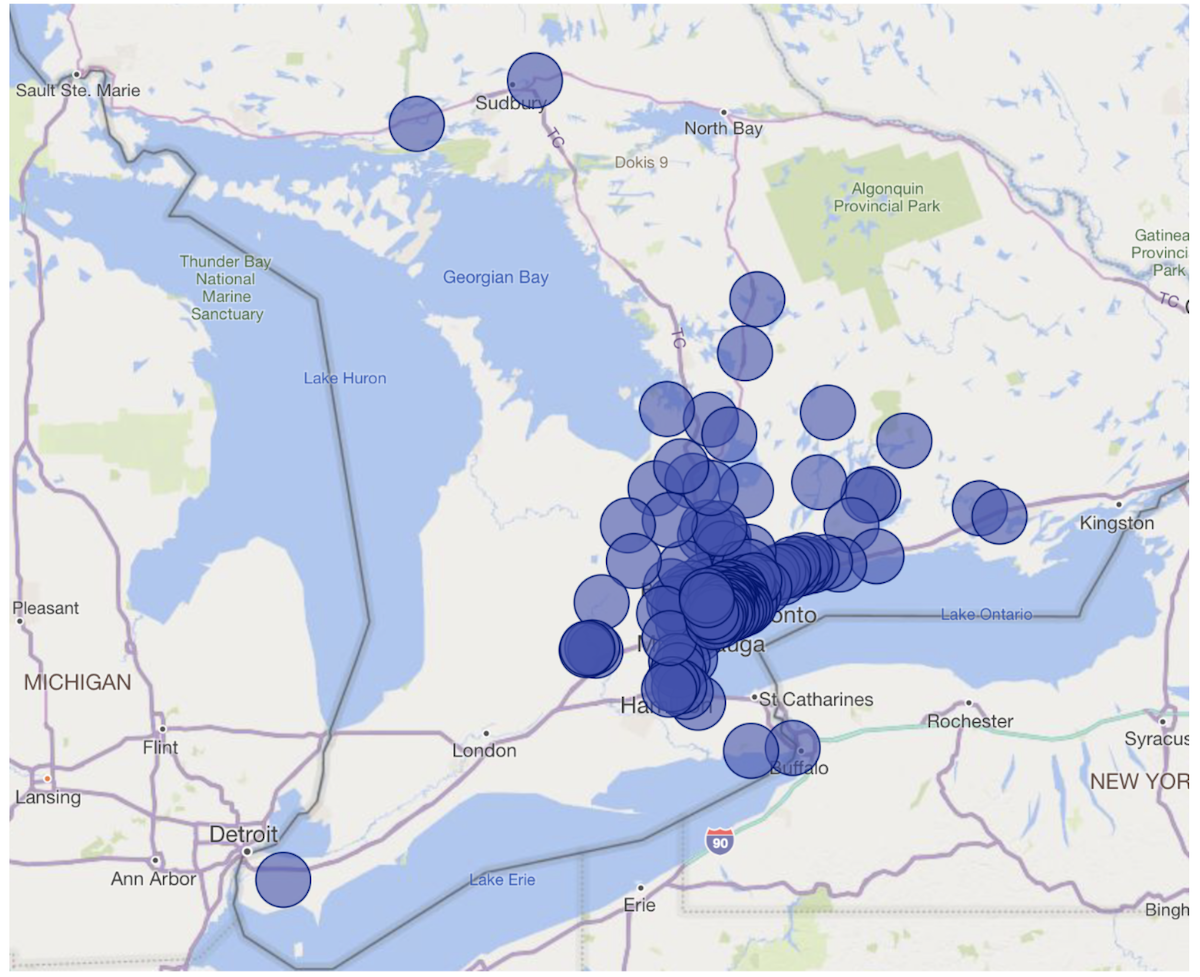
The provincewide distribution of patients presenting for Operating Room Ramp-Up After COVID Lockdown Ends—Extra Lists (ORRACLE-Xtra).

A total of 247 patients received surgery during the ORRACLE-Xtra pilot period, resulting in a 5% decrease in the total number of patients on our surgical wait list (Table 1). Of the 247 patients, 94 (38.1%) patients were female. The mean age was 5.9 (SD 4.6) years. Patients were most commonly American Society of Anesthesiologists 1 (n=209, 84.6%) and P-CATS IV priority (n=147, 59.5%), with 38.1% (n=94) being out-of-window per provincial targets. Patients travelled from across the province (Figure 2). Surgical teams from five surgical services—plastic surgery, urology, dentistry, ophthalmology, and otolaryngology (Table 2)—performed over 37 procedures (Table 3). A total of 228 hours of surgical time and 245.4 hours of anesthesia time were used. A total of 133 staff volunteered to work these extra weekend lists. The distribution of the staff working these shifts provided is shown in Multimedia Appendix 6.

**Table 1:** Patient characteristics.

		Plastics	Urology	Dentistry	Ophthalmology	Otolaryngology	Total
Age (years), mean (SD)		7.22 (5.5)	5.39 (3.4)	2.95 (1.2)	7.22 (5.0)	9.22 (5.3)	5.91 (4.6)
**Sex, n (%)**							
	Female	50 (56.3)	0 (0.0)	26 (46.6)	23 (43.4)	5 (45.4)	95 (38.5)
	Male	31 (43.7)	54 (100.0)	31 (53.4)	30 (56.6)	6 (54.5)	152 (61.5)
Cases performed, n		71	54	58	53	11	247
Blocks used, n		14	12	12	14	4	56
Surgical hours used, n		58.62	45.80	49.90	53.95	19.70	227.97
**P-CATS^a^, n (%)**							
	II^a^	0 (0.0)	0 (0.0)	0 (0.0	1 (1.9)	0 (0.0)	1 (0.4)
	III	1 (1.4)	0 (0.0)	3 (5.2)	3 (5.7)	0 (0.0)	7 (2.8)
	IV	11 (15.5)	39 (72.2)	55 (94.8)	38 (71.7)	4 (36.4)	147 (59.5)
	V	57 (80.3)	6 (11.1)	0 (0.0)	11 (20.8)	0 (0.0)	74 (30.0
	VI	2 (2.8)	9 (16.7)	0 (0.0)	0 (0.0)	7 (63.6)	18 (7.3)
**ASA^b^, n (%)^c^**							
	1	62 (88.6)	51 (94.4)	51 (87.9)	35 (66.0)	9 (81.8)	208 (84.6)
	2	8 (11.4)	3 (5.6)	5 (8.6)	17 (32.1)	1 (9.1)	34 (13.8)
	3	0 (0.0)	0 (0.0)	2 (3.4)	1 (1.9)	1 (9.1)	4 (1.6)
**Provincial WTIS^d,e^**							
	Within target	57 (80.3)	14 (25.9)	52 (89.7)	22 (41.5)	8 (72.7)	153 (61.9)
	Beyond target	14 (19.7)	40 (74.1)	6 (10.3)	31 (58.5)	1 (27.3)	94 (38.1)

^a^P-CATS: Paediatric Canadian Access Targets for Surgery.

^b^ASA: American Society of Anesthesiologists.

^c^Missing data: plastics n=1.

^d^WTIS: Wait Time Information System.

^e^Takes into consideration dates affecting readiness to treat (n=25).

**Table 2:** Surgical services and cases performed during Operating Room Ramp-Up After COVID Lockdown Ends—Extra Lists (ORRACLE-Xtra).

		Plastics	Urology	Dentistry	Ophthalmology	Otolaryngology	Total, n
Cases performed, n (%)		71 (28.7)	54 (21.9)	58 (23.5)	53 (21.5)	11 (4.5)	247
Surgical blocks used, n (%)		14 (24.6)	12 (21.1)	12 (22.8)	14 (24.6)	4 (7.0)	56
Surgical hours, n (%)		58.6 (25.7)	45.8 (20.0)	49.9 (21.9)	54.0 (23.7)	19.7 (8.6)	228.0
**Postoperative destination, n (%)**							
	Home	71 (100.0)	54 (100.0)	58 (100.0)	53 (100.0)	7 (63.6)	243 (98.4)
	Inpatient unit	0 (0.0)	0 (0.0)	0 (0.0)	0 (0.0)	4 (36.3)	4 (1.6)
Room turnover time (minutes), mean (SD)		21.5 (13.9)	21.7 (14.0)	22.5 (13.8)	27.1 (14.1)	25.4 (12.0)	23.1 (13.8)

**Table 3:** Procedures performed during the Operating Room Ramp-Up After COVID Lockdown Ends—Extra Lists (ORRACLE-Xtra) pilot program.

Procedure			Cases, n (%)
**Plastics**			
	Amputation sixth digit/polydactyly excision	6 (8.5)	
	Coleman fat transfer/fat injection	2 (2.8)	
	Cyst/lesion/skin tag excision	30 (42.3)	
	Duplicated digit reconstruction	1 (1.4)	
	Excisional biopsy	3 (4.2)	
	Hemagioma/mixed capillary and lymphatic malformation excision	4 (5.6)	
	Nervus excision	2 (2.8)	
	Plate and screw removal	1 (1.4)	
	Scar tissue revision	4 (5.6)	
	Setback otoplasty (5 bilateral, 1 unilateral)	6 (8.5)	
	Subungual exostosis excision	1 (1.4)	
	Tongue tie release	1 (1.4)	
	Trigger finger/thumb release	10 (14.1)	
**Urology**			
	Hydrocele repair	7 (13.0)	
	Orchidopexy	45 (83.3)	
	Orchiectomy	1 (1.9)	
	Penoplasty	1 (1.9)	
**Dentistry**			
	Dental extraction	1 (1.7)	
	Dental extraction and restoration	32 (55.2)	
	Dental restoration	25 (43.1)	
**Ophthalmology**			
	Botulinum injection	1 (1.9)	
	Cataract extraction	2 (3.8)	
	Conjunctival biopsy	3 (5.7)	
	Corneal crosslinking/revision	4 (7.5)	
	Entropion repair	2 (3.8)	
	Electroretinogram/retcam/fluorescein angiogram	6 (11.3)	
	Eyelid dermoid excision	1 (1.9)	
	Myectomy	1 (1.9)	
	Nystagmus surgery	1 (1.9)	
	Orbital dermoid cyst excision	3 (5.7)	
	Ptosis repair	4 (7.5)	
	Strabismus repair, rectus recession (6 bilateral, 8 unilateral)	14 (26.4)	
	Tear duct probe	11 (20.8)	
**Otolaryngology**			
	Cochlear implant (1 bilateral, 2 unilateral)	3 (27.3)	
	Fess, polypectomy, maxillary enterostomy, ethmoidectomy, sphenoidotomies, frontal, sinusotomy (bilateral)	1 (1.9)	
	Tympanoplasty	7 (63.6)	

### Outcome Measures

**Figure 3:**
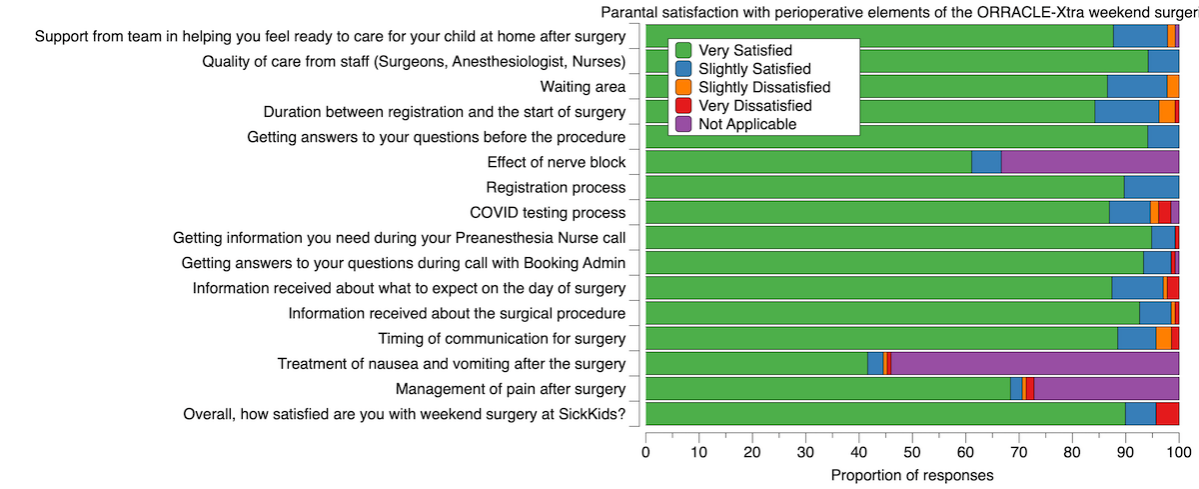
Parental experiences and satisfaction from Operating Room Ramp-Up After COVID Lockdown Ends—Extra Lists (ORRACLE-Xtra).

Outcome measures were met or exceeded in almost all instances (Table 4). Over 98% (247/250) of planned cases were completed. Preanesthesia fasting violations occurred at just 1.2% (target <15%) and lower than the concurrent monthly rate of 3.5%. Over 95.1% (235/247) of cases were completed in less than 100 minutes of surgical time (target >90%). Over 63% of blocks ended early (target 5%). In addition, 66.7%, (165/247) of surgical cases started on time (target of 85%). The mean room turnover time was 23.1 minutes (target of 31 minutes). Overall parental satisfaction was 95.8% (110/121 parent/guardian respondents; Figure 3). Challenges identified in the preprocedure areas from parental surveys led to immediate changes outlined in Multimedia Appendix 3. Of the 81 staff, 64 (79%) responding to the staff survey reported satisfaction from working on the weekend. All staff mentioned some concerns of potential burnout from working on the weekend.

**Table 4:** Process and outcome measures from the Operating Room Ramp-Up After COVID Lockdown Ends—Extra Lists (ORRACLE-Xtra) pilot program.

Measure	Expected	Actual
Completed cases, n	250	247
Scheduled cases completed (%)	97	95.74
Cancelled cases (%)	3	4.26
Cases under 100 minutes (%)	90	95.5
Preanesthesia fasting violation (%)	15	1.21
On-time starts (8 AM; %)	85	66.67
On-time starts (8:15 AM; %)	90	94.74
Operational block use (%)	75	79.17
Early block finish (%)	5	63.16
Late block finish (%)	10	1.75
Timely turnover (%)	90	88.66
Parental satisfaction (%)	80	95.87
Staff satisfaction (%)	80	79.01

## Discussion

We have shown that it is possible to establish a highly efficient weekend surgical day care model in a tertiary care pediatric hospital that can run alongside a regular weekend emergency service without interruption. ORRACLE-Xtra either met or exceeded all operational targets with low cancellation rates or preanesthesia fasting violations [16]. In addition, we found that case throughput per list and operational efficiencies were superior to those seen during the weekdays. As such, we believe that this pilot program lends itself favorably to being a sustainable model for health care delivery in the future.

The short surgical time and subsequent rapid turnover time were quicker than comparable lists during the week. However, this seemingly positive finding led to unintended consequences. The preoperative processes of each surgical list were broadly based on typical timings seen during weekday surgery. However, due to more rapid OR turnovers experienced on the weekend, the OR called subsequent patients on each list sooner than planned yet were not ready due to incomplete preoperative check-ins and fasting status. Using DMAIC, we were able to identify this problem early in the program and address it by using a preoperative flow coordinator to ensure timelier check-in of patients, particularly during the early part of the day when congestion was at its peak. As a result, predicted surgical booking times were adjusted in line with actual times, and more cases were scheduled for each list. Standard preanesthesia fasting times were also extended to account for a faster turnover of surgical cases.

In addition, due to the unexpected rapid OR throughput, the PACU was filled with postoperative patients. On occasion, the PACU could not accept subsequent patients who had completed surgeries resulting in further delays in subsequent surgeries on the list. We addressed this problem by altering the PACU staffing model to ensure more nursing availability earlier in the day to facilitate receiving more children at one time.

As operational efficiencies improved throughout this pilot program, many surgical lists finished well before the planned end-of-day allowing the surgical and nursing team to leave early (in contrast to our typical weekday workflow of reassigning teams to other areas). This had the effect of improving team morale, as reported on our provider satisfaction survey. We considered this an important finding, particularly as all staff joined the program voluntarily, and as such, we believe it helped stimulate further sign-up for future weekend lists. In addition, similar efficiencies could be realized on weekdays following similar staffing and scheduling approaches.

We also found that we could not schedule as many out-of-window cases as planned (only 38% of cases out-of-window). This meant that the overall out-of-window rate on our overall wait list did not appreciably change, given the small pilot and difficulties in bringing such children for surgery. Many families, already having waited a long time for surgery, were now reluctant to attend for surgery during the ongoing pandemic and elected to postpone until the pandemic was over. Alternatively, we found that those children who had participated in a primary assessment during the pandemic and were listed for surgery (in-window) were more willing to accept a surgical date [2]. This could be explained in part to a different risk tolerance for scheduled care during this unprecedented time. Further efforts need to be made to ensure equity of access to provide care for those children who have already experienced excessively long surgical wait times.

Over 95% of parents were satisfied with the care provided over the weekend with consistency across all surgical services. As a tertiary institution, our patient catchment area is spread across the entire province of Ontario and therefore is a sizable geographical area. However, the weekend surgeries did not appear to be a barrier to access for weekend surgery as patients from areas that are 6- to 8-hour drives away presented for a weekend surgery. Indeed, families who travelled from outside of the Greater Toronto Area commented that the hospital was more accessible on the weekend due to reduced transit time and reduced need for caregivers to take time away from work. Over 79% of staff reported being satisfied with working on the weekend. Working in this program on the weekends gave teams a sense of accomplishment and community by helping with the surgical backlog. However, some negative impact on workload and well-being was reported by nursing staff, particularly given that weekend work was in addition to the regular work schedule and thus impacted work-life balance. Administrators will need to carefully consider the overall effect of increased service from a finite pool of health care providers, with a focus on well-being.

An added benefit of using the DMAIC process to run a well-defined pilot scheduled weekend surgical project was that we were able to identify several critical improvements in service delivery. These improvements could be implemented on regular weekdays and lead to more substantial reductions in the surgical backlog:

- Fixed care teams using a designated anesthesiologist, surgeon, OR nursing, PACU nursing access, and support staff for each room improved room flow, efficiency, and throughput.
- The concept of the entire team being free to finish their shifts on completion of the list was an incentive for efficient case turnover.
- Performing more day-case surgery on the weekends may allow increased access for more medically complex surgical cases during the weekday, thus benefiting all surgical divisions and patients under the care of perioperative services.
- When provided more human resources, it will be possible to leverage empty OR complexes and unused inpatient capacity on the weekends. This allows us to scale the program to offer an even greater scope and volume of surgical activity to reduce the surgical wait list and backlog more rapidly.

In conclusion, through the ORRACLE-Xtra pilot program, we have shown that hospitals impacted by COVID-19 can use targeted MoH funding to reduce the surgical backlog associated with the COVID-19 pandemic via the use of innovative models of service delivery. In addition, sustained institutional funding to expand the perioperative workforce is critical to achieving more meaningful reductions in wait times for scheduled surgeries over the longer term. Our institution and other pediatric institutions may find the information herein helpful for regular weekday work and future pandemics.

## Appendix

Multimedia Appendix 1 Composition of the Operating Room Ramp-Up After COVID Lockdown Ends—Extra Lists (ORRACLE-Xtra) Steering Committee.

Multimedia Appendix 2 Operating Room Ramp-Up After COVID Lockdown Ends—Extra Lists (ORRACLE-Xtra) patient selection criteria for weekend elective surgery.

Multimedia Appendix 3 Decisions made throughout the 12-week pilot phase.

Multimedia Appendix 4 Schedule of meetings held throughout the 12-week pilot of Operating Room Ramp-Up After COVID Lockdown Ends—Extra Lists (ORRACLE-Xtra).

Multimedia Appendix 5 Process and outcome measures for Operating Room Ramp-Up After COVID Lockdown Ends—Extra Lists (ORRACLE-Xtra).

Multimedia Appendix 6 Characteristics of staff volunteering for Operating Room Ramp-Up After COVID Lockdown Ends—Extra Lists (ORRACLE-Xtra).

## Abbreviations

DMAIC: define, measure, analyze, improve, control
MoH: Ministry of Health
OR: operating room
ORRACLE-Xtra: Operating Room Ramp-Up After COVID Lockdown Ends—Extra Lists
PACU: postanesthesia care unit
P-CATS: Paediatric Canadian Access Targets for Surgery
SQUIRE 2.0: Revised Standards for Quality Improvement Reporting Excellence
