# Supplementary material for: A Canadian Weekend Elective Pediatric Surgery Program to Reduce the COVID-19–Related Backlog: Operating Room Ramp-Up After COVID-19 Lockdown Ends—Extra Lists (ORRACLE-Xtra) Implementation Study
Source: JMIR Perioper Med. 2022 Mar 15;5(1):e35584. doi: 10.2196/35584 (PMC8929408; doi:10.2196/35584)
Supplement: Multimedia Appendix 1 [file periop_v5i1e35584_app1.docx]

Supplementary Table 1 - Composition of ORRACLE-Xtra Steering Committee

| Name | Role |
| --- | --- |
| Clyde Matava | Anesthesiologist, ORRACLE-Xtra Program Co-lead |
| Simon Kelley | Surgeon, ORRACLE-Xtra Program Co-lead |
| Andrea Sepa | Operating Room Senior Manager |
| Angela Domingues | Post-Anaesthesia Care Unit Manager |
| Lisa Pendergast | Perioperative Services Clinical Director |
| Julianne Godden | Perioperative Services Business Operations Manager |
| Andrew Jarvis | Epic Application Specialist |
| Jeannette So | Perioperative Services Project Coordinator |
| RJ Williams | Anaesthesia Clinical Research Project Assistant |
