# Supplementary material for: A Canadian Weekend Elective Pediatric Surgery Program to Reduce the COVID-19–Related Backlog: Operating Room Ramp-Up After COVID-19 Lockdown Ends—Extra Lists (ORRACLE-Xtra) Implementation Study
Source: JMIR Perioper Med. 2022 Mar 15;5(1):e35584. doi: 10.2196/35584 (PMC8929408; doi:10.2196/35584)
Supplement: Multimedia Appendix 2 [file periop_v5i1e35584_app2.docx]

Supplementary Table 2. Patient Selection Criteria for ORRACLE-Xtra weekend elective surgery.

| Patient Criteria for ORRACLE-Xtra |
| --- |
| Medically stable ASA 1 or 2 |
| Ambulatory procedures or investigations under anesthesia (Short stay patients may be able to be accommodated in Saturday lists) |
| No logistic challenges with equipment/medication/ |
| No requirement for Acute Pathology Services (i.e. no requirement for pathologist to handle/evaluate fresh tissue samples) |
| No need for preop investigations other than MANDATORY COVID testing |
| No airway issues (vent, trach, OSA) |
| No diabetes requiring Endocrine orders |
| No sickle cell disease |
| No known cardiac disease |
| No Plan My Trip/complex behavioural issues |
| No potential for prolonged recovery/unplanned beds |
| No pre-term babies < 50 weeks post conceptual age corrected or term babies < 44 weeks |
| No Haematology patients |
