# Supplementary material for: A Canadian Weekend Elective Pediatric Surgery Program to Reduce the COVID-19–Related Backlog: Operating Room Ramp-Up After COVID-19 Lockdown Ends—Extra Lists (ORRACLE-Xtra) Implementation Study
Source: JMIR Perioper Med. 2022 Mar 15;5(1):e35584. doi: 10.2196/35584 (PMC8929408; doi:10.2196/35584)
Supplement: Multimedia Appendix 3 [file periop_v5i1e35584_app3.docx]

Decisions made throughout the 12-week pilot phase

| Week | Communication Mode | Risk or Opportunity Identified | Actions and Solutions |
| --- | --- | --- | --- |
| -3 | Virtual meeting | Humber offered OR time for SickKids patients on waitlist | - Convene Humber working group and initiate workflow planning and expedition of temporary credentialling for surgeons |
| -1 | Email | Humber stops elective surgery due to rising COVID-19 cases and can no longer accommodate SickKids patients from waitlist | - Exclude Humber as project site and focus weekend surgery at SickKids |
| 1 | Virtual meeting | Could not get approval from CHEO for batch job to administer the patient satisfaction survey via Epic by first weekend | - Epic team to add families from first weekend to batch job for automatically emailing link to survey once approval obtained |
| 2 | Virtual meeting | All blocks on first weekend ended early:   - First weekend intentionally underbooked to allow for evaluation of workflows and ensure on-time finishes - Most teams collectively decided to forgo lunch break | - Consensus from project team to add cases to blocks - Communicate changes to OR teams and admins to incrementally book additional cases per block |
| 2 | Virtual meeting | Inpatient director identified capacity to accommodate some same-day admit cases | - Work with Otolaryngology to schedule two patients requiring one day admission for upcoming Saturday |
| 2 | Virtual meeting | Dedicated staff assigned to each room facilitated patient flow and turnover through PACU | - Work with PACU team to plan weekend staffing schedule around new model for elective blocks |
| 2 | Virtual meeting | Families waited a long time in pre-op area:   - Families asked to arrive at same times as weekday surgery to prevent delays, but surgeries started half an hour later | - Consensus from project team to revise arrival times to 7AM for morning cases and 10AM for afternoon cases - Communicate update to OR teams and circulate revised patient criteria and surgical instructions to booking admins |
| 2 | Virtual meeting | Pathology technician was called in for a biopsy | - Revise patient criteria to explicitly exclude patients requiring acute pathology services and circulate revised patient criteria to surgeons and booking admins |
| 2 | Virtual meeting | One family arrived earlier than anticipated because their previous surgeries required early arrival time and there was no staff at registration and pre-op area | - Revise patient criteria and ask admins to communicate to families 7AM arrival time |
| 2 | Virtual meeting | Patient satisfaction survey cannot be automatically sent via Epic due to system constraints | - Create survey in REDCap - Send families link to survey every Monday, including families from the first weekend |
| 2 | Virtual meeting | Some families have refused consent to receive surveys for research not directly related to weekend surgery | - Check consent in patient chart before emailing satisfaction survey to families |
| 2 | Email | Email addresses not available in Epic for all families, and emails not consistently documented in same section of patient chart | - Ask registration clerks to collect emails from families on day of surgery and enter into designated section of patient chart |
| 3 | Email | There was a shortage of gowns and flannel blankets and more supplies had to be ordered on both Saturday and Sunday | - Provide additional communication to Supplies that there will be elective surgeries on weekends until end of March and to adjust stock accordingly - Provide POCU attendants with contact for Supplies and recommend the contact also be left at work station |
| 3 | Email | There was a shortage of cribs and wheelchairs in the OR and pre-op area, and the cribs were placed by the pre-op outer hallway, which blocked the way into the OR for families with strollers and wheelchairs for sedated patients | - Provide additional communication to Transport that there are elective surgeries on weekends until end of March and to adjust stock accordingly - Move cribs out of the pre-op outer hallway |
| 4 | Virtual meeting | Anesthesia identified they still had capacity to increase number of cases performed per block, but rapid speed of cases overwhelming PACU | - Work with Anesthesia to modulate patient flow to PACU - Continue to review PACU staff feedback and adjust as needed |
| 4 | Virtual meeting | Clinic informed one family to arrive 2 hours early for surgery:   - No staff at registration or pre-op - Long wait for surgery | - Project team agree on new arrival times: 6:45AM for first case, 7:00AM for other morning cases, and 10:00AM for all cases after noon - Communicate update to OR teams and circulate surgical instructions to booking admins, highlighting that pre-op will not be open before 6:45AM |
| 4 | Virtual meeting | Four families went to the parents’ waiting room after registration instead of to pre-op and later found by a nurse, resulting in delays in pre-op | - Create signs for parents’ waiting room door and the OR front doors with directions to pre-op |
| 4 | Virtual meeting | One additional staff in pre-op improved patient flow and set-up | - Coordinate staffing schedule to have one additional staff to help out at pre-op at patient arrival times |
| 4 | Virtual meeting | Additional staff to help organize and manage patients reduced confusion and congestion at registration and pre-op | - Train two staff as flow coordinators to help at registration, pre-op, and PACU for weekend elective surgery |
| 4 | Virtual meeting | Two patients who underwent surgery were ASA 3, which did not meet criteria of ASA 1 or 2 for weekend elective surgery | - Follow up to determine why patients were scheduled - Provide additional communication to admins on importance of following patient criteria when scheduling surgery on weekends |
| 4 | Virtual meeting | Feedback from PACU staff and patient surveys that some patients were still groggy when discharged   - Cases completed so quickly that patients still experiencing effects of anesthetic | - Project team to monitor |
| 4 | Email | Low response rate to patient satisfaction survey | - Update survey administration schedule to include one follow-up reminder on Wednesdays |
| 5 | Virtual meeting | Patient compliant with NPO timeline, but list moved so quickly that OR was ready before scheduled time and surgeon made decision to cancel case rather than wait for scheduled start time | - Project team and Anesthesia agree to extend NPO timeline from 3 to 4 hours for weekend surgery, with pre-op nurse consulting Anesthesia for exceptions for clear liquids as needed - Communicate update to OR teams and booking admins |
| 6 | Virtual meeting | More than 50% of cases completed after 5 weeks were out of window, even taking into consideration DARTS:   - Families choosing to defer surgery due to pandemic - Given short timeframe to schedule cases, easier to contact patients recently seen in clinic to ensure block is filled - Due to strict patient criteria, surgeons are running out of eligible patients on waitlist - Some surgeons operating on weekends are newer staff and do not have long waitlists | - Follow up with division heads/department chiefs and booking admins on case selection process |
| 9 | Email | Received additional funding to continue weekend surgery through to June, but Anesthesia identified that all staff have volunteered for at least one weekend block and sign-up is declining, uncertain of adequate staffing beyond March | - Work with Anesthesia to verify staffing needs until end of March - Revise block allocation schedule for April to June based on Anesthesia staffing - Develop and administer staff satisfaction survey; use results to inform future planning |
