# Supplementary material for: A Canadian Weekend Elective Pediatric Surgery Program to Reduce the COVID-19–Related Backlog: Operating Room Ramp-Up After COVID-19 Lockdown Ends—Extra Lists (ORRACLE-Xtra) Implementation Study
Source: JMIR Perioper Med. 2022 Mar 15;5(1):e35584. doi: 10.2196/35584 (PMC8929408; doi:10.2196/35584)
Supplement: Multimedia Appendix 4 [file periop_v5i1e35584_app4.docx]

Schedule of meetings held throughout the 12-week pilot

| Virtual Meeting | Date |
| --- | --- |
| Kick-off | December 14, 2020 |
| Humber-SickKids waitlist | December 14, 2020 |
| OR operations team | December 15, 2020 |
| Admin support | December 15, 2020 |
| Coordination of SickKids patients at Humber | December 16, 2020 |
| Epic team | December 17, 2020 |
| Epic team on patient satisfaction survey | December 17, 2020 |
| Team update | December 18, 2020 |
| QI application | December 21, 2020 |
| Project management and implementation | December 23, 2020 |
| Surgical booking admins | January 4, 2021 |
| Epic and workflows | January 6, 2021 |
| Patient satisfaction survey | January 7, 2021 |
| Epic team on patient satisfaction survey | January 7, 2021 |
| Team update | January 14, 2021 |
| Team update | January 19, 2021 |
| Leads update | January 25, 2021 |
| Team update | February 4, 2021 |
| Team update | February 8, 2021 |
| Team update | February 25, 2021 |
| Weekend surgery dashboard | February 26, 2021 |
| Data and dashboard update | March 10, 2021 |
| Team update | March 17, 2021 |
| Dashboard update | March 18, 2021 |
| Data for manuscript | May 10, 2021 |
