# Supplementary material for: A Canadian Weekend Elective Pediatric Surgery Program to Reduce the COVID-19–Related Backlog: Operating Room Ramp-Up After COVID-19 Lockdown Ends—Extra Lists (ORRACLE-Xtra) Implementation Study
Source: JMIR Perioper Med. 2022 Mar 15;5(1):e35584. doi: 10.2196/35584 (PMC8929408; doi:10.2196/35584)
Supplement: Multimedia Appendix 5 [file periop_v5i1e35584_app5.docx]

Supplementary Table 7 Process and Outcome Measures

| Measure | ORRACLE -Xtra | Target |
| --- | --- | --- |
| Completed Cases | 247 | 250 |
| Scheduled Cases Completed | 95.7% | 97.0% |
| Cancelled Cases | 4.3% | 3.0% |
| Cases under 100 minutes | 95.5% | 90.0% |
| NPO Violation | 1.2% | 15.0% |
| On-Time Start (8:00am) | 66.7% | 85.0% |
| On-Time Start (8:15am) | 94.7% | 90.0% |
| Operational Block Utilization | 79.2% | 75.0% |
| Early Block Finish | 63.2% | 5.0% |
| Late Block Finish | 1.8% | 10.0% |
| Rapid Turnover (<25 min) | 88.7% | 90.0% |
| Care-giver Satisfaction | 95.9% | 80.0% |
| Provider Satisfaction | 79.0% | 80.0% |
