# Supplementary material for: A Canadian Weekend Elective Pediatric Surgery Program to Reduce the COVID-19–Related Backlog: Operating Room Ramp-Up After COVID-19 Lockdown Ends—Extra Lists (ORRACLE-Xtra) Implementation Study
Source: JMIR Perioper Med. 2022 Mar 15;5(1):e35584. doi: 10.2196/35584 (PMC8929408; doi:10.2196/35584)
Supplement: Multimedia Appendix 6 [file periop_v5i1e35584_app6.docx]

Supplementary Table 6 Characteristics of Staff volunteering

| Role | Staff (N) | Mean (h) | Median (h) | Range (h) |
| --- | --- | --- | --- | --- |
| Surgeon | 22 | 18.1 | 13.3 | 3.3-55.0 |
| Surgical Fellow | 6 | 18.9 | 18.8 | 5.0-29.0 |
| Anesthesiologist | 36 | 11.1 | 12.5 | 4.8-15.8 |
| Peri-Anesthesia Nursing | 22 |  |  |  |
| Intra-operative Nursing | 33 | 34.5 | 22.5 | 7.5-142.5 |
| POCU Attendant | 13 | 86.5 | 60.0 | 7.5-180.0 |
| Flow Coordinators | 1 | 6.6 | 6.5 | 138.5 |
| Registration/Clerks | 3 | 7.0 | 7.0 | 441.0 |
